# Ecofriendly Green Synthesis of Copper (II) Oxide Nanoparticles Using *Corchorus olitorus* Leaves (Molokhaia) Extract and Their Application for the Environmental Remediation of Direct Violet Dye via Advanced Oxidation Process

**DOI:** 10.3390/molecules28010016

**Published:** 2022-12-20

**Authors:** Reham O. Aljedaani, Samia A. Kosa, Mohamed Abdel Salam

**Affiliations:** Department of Chemistry, Faculty of Science, King Abdulaziz University, P.O. Box 80200, Jeddah 21589, Saudi Arabia

**Keywords:** CuO, green preparation, direct dye, environmental remediation, kinetics

## Abstract

In this research, copper (II) oxide nanoparticles were prepared by an ecofriendly green method using the extract of *corchorus olitorus* leaves (Molokhaia) as a surfactant, capping and anti-agglomeration agent. The ecofriendly green CuO NPs were characterized using different chemical and physical techniques and the results confirmed the formation of monoclinic tenorite CuO nanoparticles with an average particle size of 12 nm and BET surface area of 11.1 m^2^/g. The eco-friendly green CuO NPs were used in environmental remediation for the efficient catalytic degradation of direct violet dye via advanced oxidation process (AOP) in presence of H_2_O_2_. The impact of AOP environmental parameters affecting the degradation process was investigated. Moreover, the catalytic degradation of the direct violet dye using the ecofriendly green CuO NPs was studied kinetically and thermodynamically and the results showed that the catalytic degradation process agreed well with the pseudo-second-order kinetic model and the process was spontaneous and endothermic in nature. Finally, high catalytic degradation of the direct violet dye was observed when the eco-friendly prepared green CuO NPs were placed in real water samples.

## 1. Introduction

Water pollution has been a recurrent environmental issue and global challenge, particularly due to the exceptionally increasing rate of anthropogenic activities, such as population expansion, industrialization and agricultural practices, which consequently make the issue of water quantity, quality and availability a main concern in many parts of the world [1]. The efforts of research scientists, decision-makers and legislators focus on the introduction of more rigorous methods for effective environmental remediation, as well as the introduction of new laws to control environmental pollution. Conversely, the application of synthetic dyes became more common in the production of clothes, furniture and plastic products instead of natural dyes, but unfortunately these dyes are toxic and carcinogenic, affecting aquatic and human life [2]. Discouragingly, conventional water treatment methods such as coagulation, sedimentation, filtration, disinfection, decontamination and desalination are chemically and operationally intensive, which mainly require large systems, infrastructure and engineering expertise, making the treatment time-consuming, costly and ineffective when immediate demand arises. Currently, the search for and development of new robust procedures with high efficiency for the remediation of polluted water are mandatory and urgent. In this scenario, nanotechnology is found to be a promising route for the development of an effective procedure for the treatment of polluted water, water pollution prevention, detection and water quality monitoring [3,4]. This is a result of the unique and exceptional properties of nanomaterials, as the nanoscale level particles have different chemical, physical, biological, optical, magnetic and electrical properties compared to macro-particles, providing a wide range of applications [5]. Metal oxide nanoparticles such as copper oxides have become increasingly important in a wide range of nanotechnology and environmental remediation [1], environmental monitoring [6], catalysis [7,8], biological activities [9,10,11], energy [12], organic reactions [13] and nanofluid [14]. CuO NPs were synthesized using different chemical or physical procedures such as chemical vapor deposition [15], sol-gel [16], liquid phase [17], electrodeposition [18] and colloidal [19] methods. Unfortunately, the synthesis of CuO NPs using physical and chemical methods involves laborious steps with long reaction times and the use of various hazardous chemicals compounds, in addition to the generation of huge amounts of dangerous byproducts, as well as hazardous chemical waste [20]. During the last few years, more eco-friendly, biosynthesis and green procedures have been applied based on natural-based extracts of different plants, algae, fungi and bacteria as capping and anti-agglomeration agents to facilitate the formation of the CuO NPs [21,22]. Comparing plants and microorganisms, plants are more suitable as they are easily available, can be directly employed without following any complex procedure and contain bioactive substances such as terpenoids, proteins, sugar, phenolic compounds, flavonoids and alkaloids, which can easily reduce the metal ions into stable nanoparticles [23]. More importantly, the biosynthesized CuO NPs are characterized by higher activities compared with the conventionally synthesized ones [21,22,24]. Understanding this, new methods are required for the biosynthesis of CuO NPs, as it is a challenging task to find a more convenient method.

Advanced oxidation processes (AOPs) are commonly used to remove/eliminate/degrade different pollutants in contaminated water by an oxidation process in the presence of ozone (O_3_), hydrogen peroxide (H_2_O_2_) and/or UV light through reactions with hydroxyl radicals (^·^OH) [25]; therefore, AOPs are promising technologies for the elimination of dyes in solution because they are cheap, more efficient, eco-friendly and can completely degrade the pollutants to water and carbon dioxide [26,27]. Much research focuses on the application of metal/metal oxides nanoparticles and AOPs for water treatment. Metal/metal oxide nanoparticles are mainly composed of one, two, or three metals (such as Ag, Au and Pd nanoparticles) and/or metal oxides (such as ZnO, V_2_O_5_, Ta_2_O_5_, CeO_2_, MgO, CuO and TiO_2_) used for the degradation of organic pollutants in water, due to their high surface contact area and improved photolytic properties, thus being considered the best candidate for water remediation [28,29,30]. Among these, CuO is considered as a cheap, non-toxic and effective environmentally friendly catalyst and was used as an effective catalyst for AOPs in the environmental remediation of water polluted with different pollutants [31,32,33,34].

In this research, a new, simple and efficient strategy for the eco-friendly, green and robust biosynthesis of CuO nanoparticles was explored using *corchorus olitorius* leaves extract (Molokhia), one of the most common plant leaves in the region. The morphological and structural characteristics of the eco-friendly green CuO NPs were investigated and applied as a catalyst for the catalytic degradation of direct violet Dye 31 (DV31) via advanced oxidation process AOPs in presence of hydrogen peroxide.

## 2. Experimental Section

### 2.1. Materials

All chemicals were obtained from SIGMA-ALDRICH, were general-purpose reagents and were used in their original form without further purification. The leaves of *corchorus olitorus* (Molokhia) were obtained from Jeddah local market.

#### 2.1.1. *Corchorus Olitorus* Leaves Extract (Molokhia) Preparation

Fresh and healthy leaves of *corchorus olitorus* were collected from the local market in Jeddah, Saudi Arabia. The leaves were washed very well 3 times with tap water, then 100.0 g of the cleaned leaves were added to 100 mL of deionized water in a beaker. The mixture was boiled for 20.0 min, then cooled to room temperature and filtered; a clear filtrate was obtained and was used to prepare the solutions of NaOH and CuSO_4_.

#### 2.1.2. Conventional CuO NPs (CuO NPs) Preparation

An aqueous solution of copper sulfate pentahydrate (CuSO_4_.5H_2_O) (50.0 mL, 1.0 M) and the solution of NaOH (100 mL, 1.0 M) were prepared with deionized water. The NaOH solution was added drop by drop using a burette to the copper sulfate solution at 70 °C temperature under 200 rpm stirring. The black precipitate of the colloidal copper oxide was separated by centrifugation at 3500 rpm for 30 min and washed with deionized water three times, followed by acetone. The obtained product was dried at 110 °C in air atmosphere for 24 h in a regular drying oven and then calcinated in a muffle furnace at 500 °C for 60 min.

#### 2.1.3. Green CuO NPs (G CuO NPs) Preparation

Green CuO NPs were prepared according to the literature with little modification [35]. An aqueous solution of copper sulfate pentahydrate (CuSO_4_.5H_2_O) (50.0 mL, 1.0 M) and the solution of NaOH (100 mL, 1.0 M) were prepared using the *corchorus olitorus* extract. The NaOH solution was added drop by drop using a burette to the copper sulfate solution at 70 °C temperature under 200 rpm stirring. The black precipitate of the colloidal copper oxide was separated by centrifugation at 3500 rpm for 30 min and washed with deionized water three times, followed by acetone. The obtained product was dried at 110 °C in air atmosphere for 24 h in a regular drying oven and then calcinated in a muffle furnace at 500 °C for 60 min.

### 2.2. Characterization Techniques

X-ray diffraction (XRD) patterns were recorded for phase analysis and the measurement of crystallite size on an X-ray diffraction device (XRD; type Ultima-IV; Rigaku, Tokyo, Japan), which was operated at 40 mA and 40 kV by using CuK αradiation and a nickel filter in the 2θ range from 20 to 80° in steps of 0.02°, with a sampling time of one second per step. Scherrer equation was used for the calculation of crystal size of the CuO NPs. The morphology and size of the CuO NPs were examined using a scanning electron microscope (SEM; type JEOL JSM-7600F) and transmission electron microscope (TEM; type JEOL JEM-2100 operating at 200 kV, attached to a CCD camera). The XPS experiments were performed on a Kratos Axis Ultra DLD instrument equipped with a monochromatic Al Kα X-ray source (hν = 1486.6 eV) operated at a power of 75 W and under UHV conditions in the range of ∼10−9 mbar. All spectra were recorded in hybrid mode using electrostatic and magnetic lenses and an aperture slot of 300 μm × 700 μm. The survey and high-resolution spectra were acquired at fixed analyzer pass energies of 160 and 20 eV, respectively. The samples were mounted in floating mode in order to avoid differential charging. Thereafter, XPS spectra were acquired using charge neutralization. The specific surface area of the CuO NPs were estimated using the nitrogen adsorption/desorption isotherm at 77 K, by NOVA3200e (Quantachrome, Boynton Beach, FL, USA).

### 2.3. Removal Study

The advanced oxidation processes for the removal of the DV31 dye from water were performed using CuO NPs in presence of H_2_O_2_ as follows: _a_ certain amount of CuO NPs was added to 100 mL containing DV31 dye (25.0 mg/L) in the presence of H_2_O_2_, followed by magnetic stirring, and the CuO NPs were separated from the solution using a centrifuge and at the offset time. The clear filtrate was collected for the analysis of the remaining DV31 dye using ultraviolet-visible (UV–vis) spectrophotometer (UV-1650 PC, CPS-240A SHIMADZU) at 553 nm. The removal percentage (%R) and the removal capacity; q_t_ (mg/g), were calculated using the following equations:

(1) $$\%R=\frac{C_{0}-C_{t}}{C_{0}}\times 100$$

(2) $$q_{t}=\left(C_{0}-C_{t}\right)\times \frac{V}{m}$$

where C_0_ and C_t_ are the concentrations of the DV31 dye in solution (mg/L) at time t = 0 and t, respectively, V is the volume of the solution (L) and m is the mass of CuO NPs (g).

## 3. Results & Discussion

### 3.1. Characterization of CuO NPs

**Figure 1:**
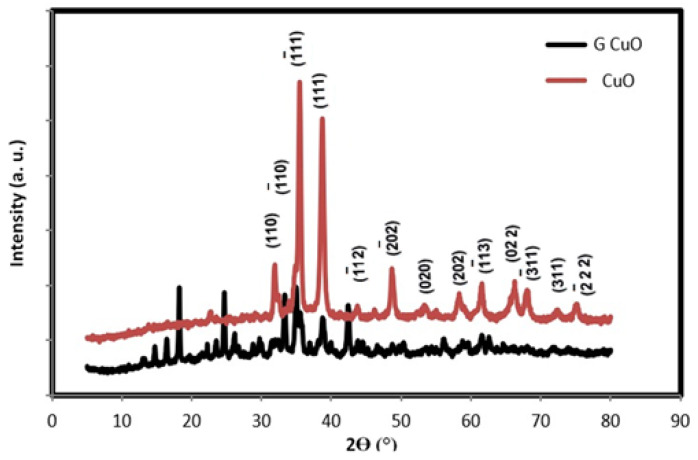
XRD pattern of different for CuO NPs and G CuO NPs.

**Figure 2:**
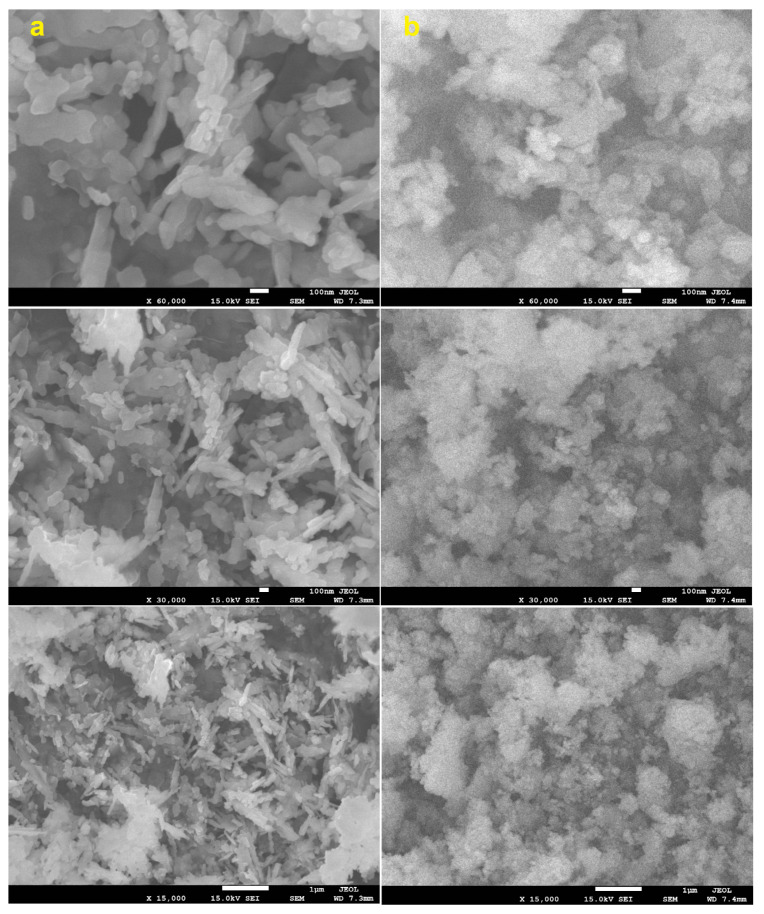
Scanning electron microscope (SEM) images for (**a**) T CuO NPs and (**b**) G CuO NPs.

**Figure 3:**
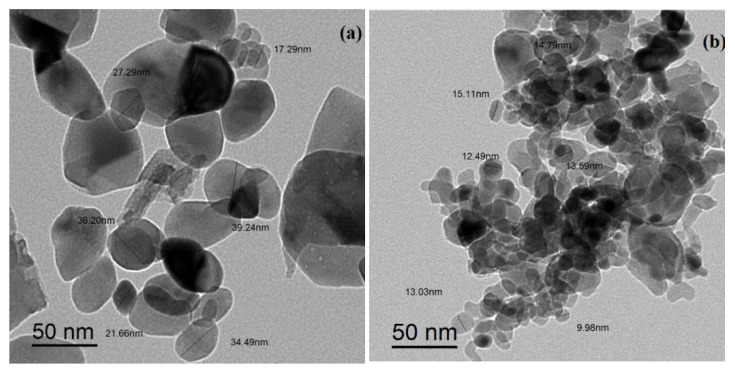
Transmission electron microscope (TEM) images for (**a**) CuO NPs and (**b**) G CuO NPs.

The morphological structure and composition of the prepared CuO NPs, conventional CuO NPs (CuO NPs) and conventional green CuO NPs (G CuO NPs), were explored using XRD, SEM, TEM, XPS and BET. The crystal structure and purity of the different CuO NPs were examined by XRD. Figure 1 shows the XRD pattern of CuO NPs, which showed the presence of characteristic peaks at 2θ values of 32.9°, 33.5°, 35.9°, 39.1°, 42.3°, 49.1°, 54.9°, 58.7°, 61.9°, 66.5°, 68.4°, 72.8° and 75.5°, related to (110), ($\bar{1}$10), ($\bar{1}$11), (111), ($\bar{1}$12), ($\bar{2}$02), (020), (202), ($\bar{1}$13), (022), ($\bar{3}$11), (311) and ($\bar{2}$22) planes for both the conventional and green CuO, respectively, which can be indexed on the basis of monoclinic tenorite CuO with the C2/c space group (JCPDs file No. 01-080-1916) [36], indicating the good crystallinity of the prepared CuO NPs and the absence of any characteristics peaks of any other phase of CuO. Moreover, there were few unidentified diffractions peaks at 2θ values less than 20°; especially for the conventional CuO, which could not be assigned to any copper-based compound and were considered as impurities. It is also observed that the intensities of the peaks were different, indicating the different degrees of crystallinity as well as the different particle sizes depending on the preparation method. The size of the crystals (D) has been calculated from the FWHM of the main peak with the Debye Scherrer formula using Equation (3):

D = K λ/β COS θ (3)

where K is constant, λ is the wavelength of X-rays employed radiation (1.54056 Å), β is corrected full width at half maximum and θ is Bragg angle, and the crystallite sizes of the CuO NPs were 14.7 nm and 14.4 nm for the T CuO NPs and G CuO NPs, respectively. Figure 2 and Figure 3 show the SEM and TEM images of the different CuO NPs. It is clear that the preparation method greatly affects the shape and dimensions of the CuO NPs as all of them featured different shapes, lengths and widths. The conventional and green CuO NPs were spherical in shape with average particle size of 36 nm and 12 nm, respectively.

**Figure 4:**
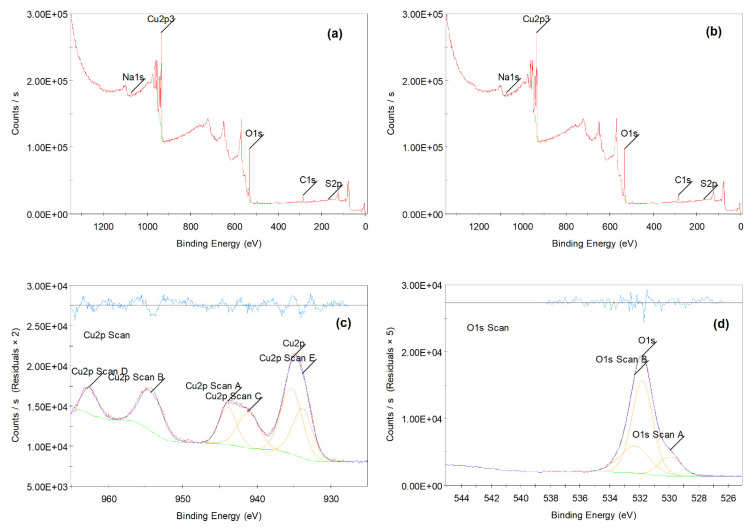
Survey or wide energy range X-ray photoelectron spectroscopy (XPS) spectra of CuO NPs (**a**), G CuO NPs (**b**) and high resolution (Core level) or narrow energy range spectra of Cu 2p (**c**) and O 1s (**d**), from the G CuO NPs.

The surface binding state and elemental speciation of the different CuO NPs were analyzed by XPS and the wide-scan XPS survey spectra are presented in Figure 4, which confirmed the presence mainly of copper (Cu) and oxygen (O). The XPS survey spectrum of CuO NPs showed sub peaks at binding energies 934.24 eV corresponding to the Cu 2p3 sub peaks corresponding to Cu^+2^ in CuO NPs; these sub peaks are also consistent with the oxygen bonds indicated as O1s at binding energies and 531 eV. Additionally, the presence of an insignificant amount of carbon and sodium within the CuO NPs sample could be attributed to the preparation process. Figure 4c shows the high-resolution spectra (core XPS spectra) of Cu 2p, which presents the main shake-up peak at the higher binding BE side of the Cu 2p_3/2_ and the increase in the binding energy of the man peak, suggesting the existence of an unfilled *Cu3d_9_* shell, confirming the presence of Cu^2+^ in the prepared samples of G CuO NPs. Figure 4d shows the high-resolution spectra (core XPS spectra) of O1s, which shows three components at 529.1 eV, 530.8 eV and 532.2 eV. The first peak at 529.1 eV could be assigned to the BE for lattice oxygen (O_L_)^2−^ in the G CuO NPs lattice, which is in good agreement with the BE of O^2−^ ion in the metal oxide sites (Cu^2+^-O^2−^). The second peak at 530.8 eV could be assigned to the BE for oxygen defects/vacancies (O_V_)^2−^ in the G CuO NPs matrix, whereas the last peak at 532.2 eV could be assigned to the BE for the adsorbed residual carbon or other surface oxygen species, which easily reacts with the G CuO NPs surface [37].

The specific surface areas of the different CuO NPs were calculated from the nitrogen gas adsorption/desorption isotherms at 77 K applying the BET equation. The BET specific surface areas were 9.4 and 11.1 m^2^/g for the T CuO NPs and TG CuO NPs, respectively. The calculated average pore diameters were 60.3 nm and 1.93 nm and the pore volume were 0.232 cm^3^/g and 0.160 cm^3^/g for the T CuO NPs and TG CuO NPs, respectively.

The above characterization results demonstrate the major effect and the significant enhancement in the CuO NPs upon the application of the *corchorus olitorus* extract which acts as a capping and stabilizing agent and prevents the agglomeration of the CuO NPs due to the presence of long-chain natural products in the plant extract [38].

### 3.2. Removal Study

**Figure 5:**
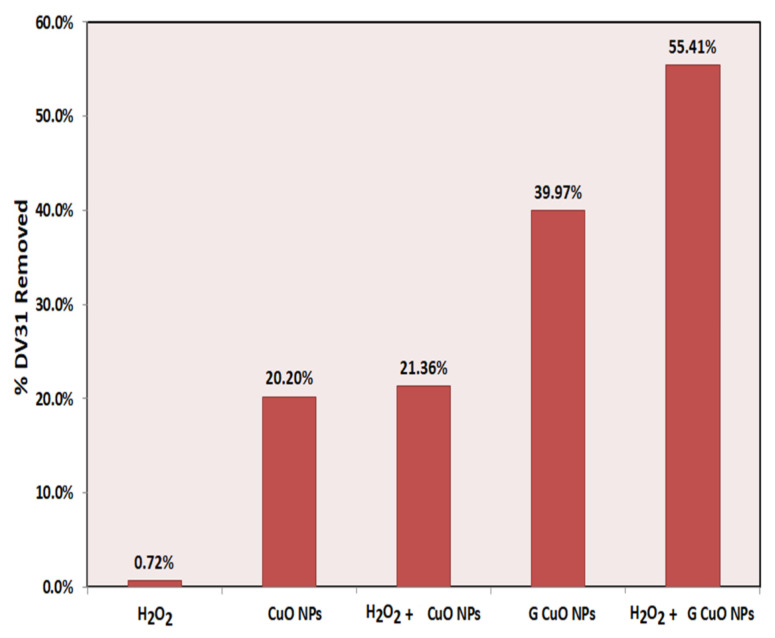
Selection of the suitable CuO NPs for the removal DV31 dye from aqueous solution. (Experimental conditions: DV31 dye concentration 25 mg/L; sample volume 100 mL; 10.0 mg CuO NPs; 1 mL H_2_O_2_; 30 min contact time; pH 8.6 and temperature at 25 °C).

In this comparative study, the removal of DV31 dye from aqueous solution was performed using the different CuO NPs synthesized through a different route (conventional and green) in the presence and absence of the hydrogen peroxide in order to select the most effective NPs, and the results are presented in Figure 5. According to the figure, hydrogen peroxide showed no effect on the catalytic degradation of the DV31 dye in the absence of CuO NPs as the removal percentage was 0.72%, whereas the CuO NPs in the absence of the hydrogen peroxide have low removal efficacies: 20.20% and 39.97%, for T CuO NPs and G CuO NPs, respectively. These removal efficacies were enhanced greatly upon the combination of both CuO NPs and hydrogen peroxide, as the removal percentage reached 21.36% and 55.41% for the hydrogen peroxide and T CuO NPs and G CuO NPs, respectively. This may indicate that CuO NPs may activate the hydrogen peroxide and produce free radicals which greatly participate in the degradation of the DV31 dye [39]. The experimental results revealed that the removal of DV31 dye was higher in the case of G CuO NPs prepared using corchorus olitorus as the capping agent by precipitation method, compared with the conventional CuO NPs. This may be attributed to the small particle size of the G CuO NPs as well as the good dispersion compared with the T CuO NPs. Accordingly, the G CuO NPs were selected for the rest of the experiments to study the removal of DV31 dye in the presence of hydrogen peroxide via AOP.

**Figure 6:**
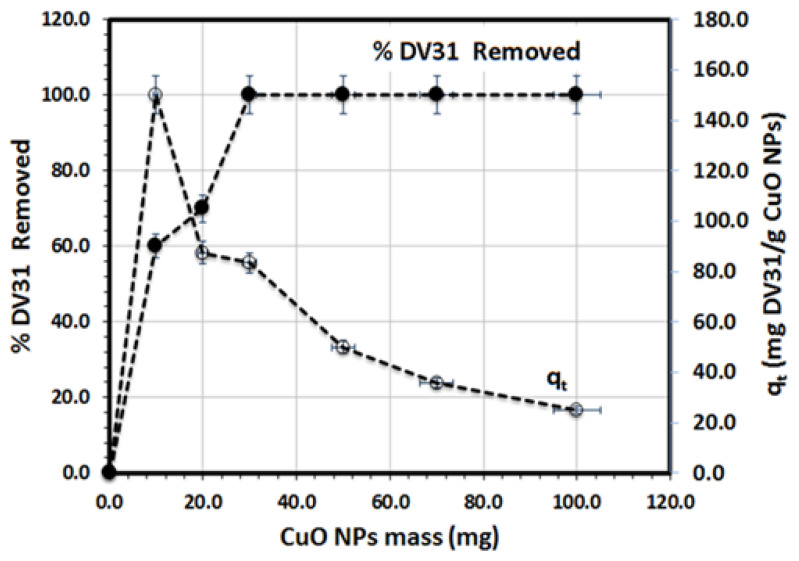
Effect of CuO NPs mass for the removal of DV31 dye from aqueous solution. (Experimental conditions: DV31 dye concentration 25 mg/L; sample volume 100 mL; 30 min contact time; 0.5 mL H_2_O_2_; pH 8.6 and temperature at 25 °C).

The effect of G CuO NPs mass on the removal of DV31 dye from aqueous solution was studied and the results are presented in Figure 6. The experimental results revealed that increasing the G CuO NPs mass greatly enhanced the removal efficiency due to the increase in the number of active sites available for the binding of the DV31 dye molecules, till it reached its complete removal of the DV31 dye using 30 mg of G CuO NPs, whereas the removal capacity was decreased linearly with increasing the G CuO NPs mass and the maximum capacity obtained was 150.25 mg DV31/g CuO NPs using 10 mg of the CuO NPs in the presence of 0.5 mL of hydrogen peroxide.

**Figure 7:**
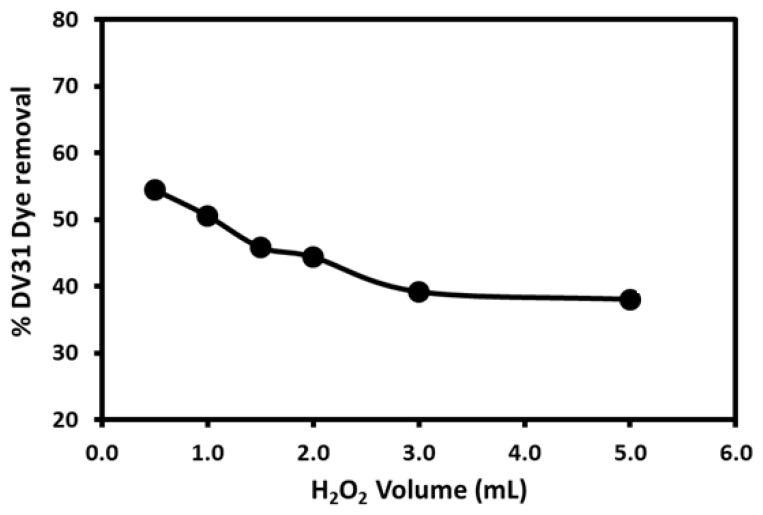
Effect of H_2_O_2_ volume on the removal of DV31 dye from aqueous solution using G CuO NPs. (Experimental conditions: DV31 dye concentration 25 mg/L; sample volume 100 mL; 10.0 mg CuO NPs; pH 8.6 and temperature at 25 °C).

The effect of the H_2_O_2_ volume on the degradation of DV31 dye in the presence of the G CuO NPs was optimized (Figure 7). It is clear that increasing H_2_O_2_ volume significantly decreased the removal efficiency of the DV31 dye, which may indicate that H_2_O_2_ is not the main reagent for the catalytic degradation of the DV31 dye molecules in water, the degradation mainly depends on the presence of CuO NPs and the H_2_O_2_ as the CuO NPs may activate the hydrogen peroxide and produce free radicals, which greatly participate in the degradation of the DV31 dye.

**Figure 8:**
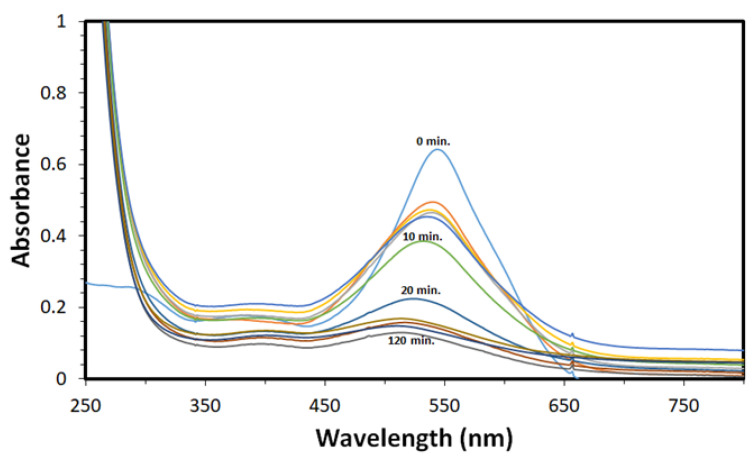
The variation of the uv-vis absorbance with time for the removal of DV31 dye from aqueous solution using G CuO NPs. (Experimental conditions: DV31 dye concentration 25 mg/L; sample volume 200 mL; 20.0 mg CuO NPs; 1 mL H_2_O_2_; pH 8.6 and temperature at 25 °C).

The contact, interaction and removal time between the pollutant such as DV31 dye and the solid catalyst such as CuO NPs is an important factor greatly affecting the removal of any pollutants from the environment. Figure 8 shows the UV-Vis spectra of DV31 dye and the variation of the absorbance with time for the removal of DV31 dye from aqueous solution using G CuO NPs.

**Figure 9:**
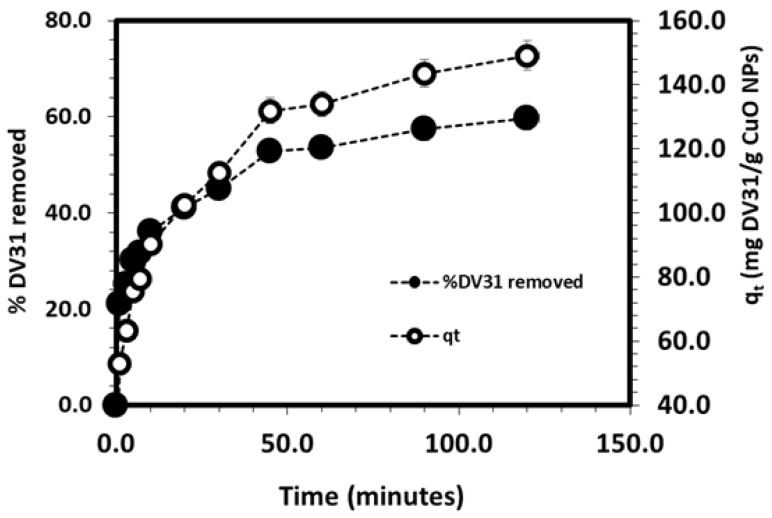
Effect of contact time on the removal of DV31 dye from aqueous solution using G CuO NPs. (Experimental conditions: DV31 dye concentration 25 mg/L; sample volume 200 mL; 20.0 mg CuO NPs; 1 mL H_2_O_2_; pH 8.6 and temperature at 25 °C).

The effect of the contact time on DV31 dye removal from model solution by CuO NPs was explored and the results are shown in Figure 9. It is noted that the percentage removal of DV31 increased within the first 30.0 min with 45% efficacy and removal capacity of 112.48 mg DV31/g CuO NPs. Increasing the reaction time was associated with an insignificant increase of the percentage removal to 60% and removal capacity of 149.15 mg DV31 dye/g CuO NPs after 120 min. According to the results, the equilibration time was assigned as 30.0 min for the rest of the experiments.

**Figure 10:**
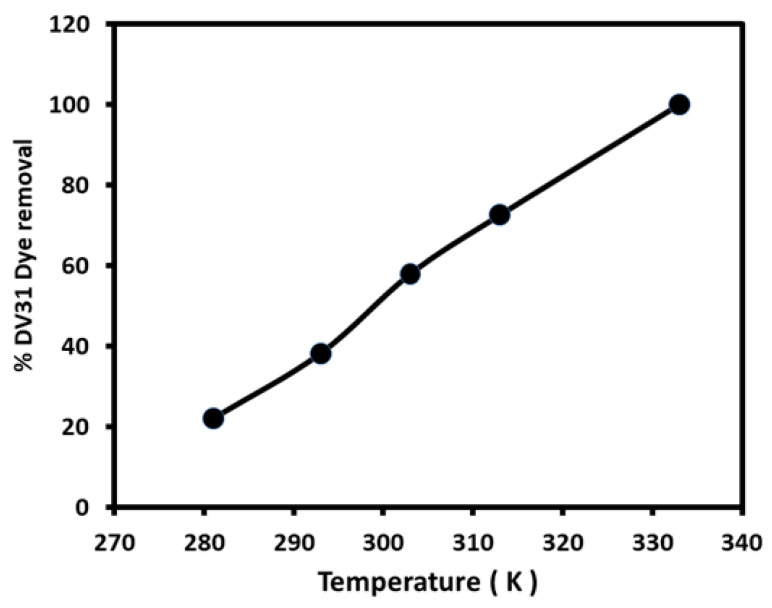
Effect of solution temperature on the removal of DV31 dye from aqueous solution using G CuO NPs. (Experimental conditions: DV31 dye concentration 25 mg/L; sample volume 100 mL; 10.0 mg CuO NPs;0.5 mL H_2_O_2_; 10 min contact time and pH 8.6).

The variation of the catalytic degradation of DV31 dye using G CuO NPs by solution temperature was studied in the presence of 0.5 mL hydrogen peroxide. According to Figure 10, raising the solution temperature correlated with a significant increase in the % DV31 dye degradation; 22.07% at 281 K to 100% at 333 K, indicating the endothermic nature of the removal process. The observed enhancement of the removal efficacy may be attributed to two main factors; the first factor may be the improvement in the diffusion of the DV31 dye molecules from the bulk solution to the G CuO NPs surface associated with rising the solution temperature owing to the decrease in the viscosity of the solution [40] and the second factor is the fact that increasing the solution temperature enhanced the degradation of the hydrogen peroxide and the production of the hydroxyl free radicals [41], which reacted with CuO NPs and degraded the DV31 dye molecules.

**Figure 11:**
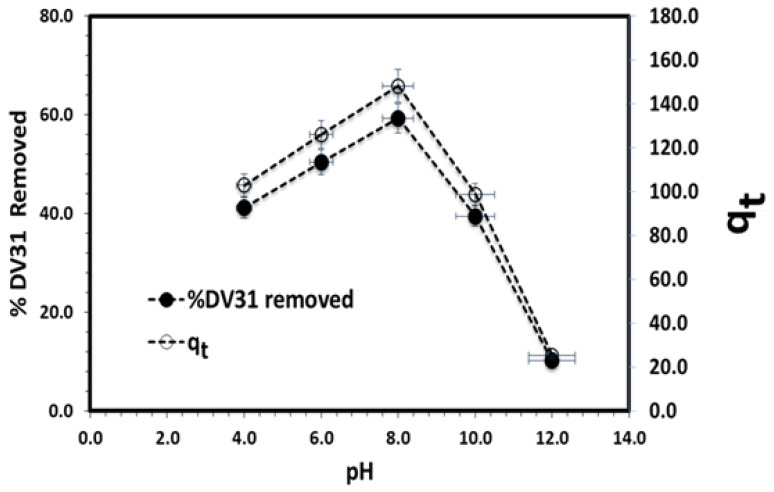
Effect of solution pH on DV31 dye removal from an aqueous solution by CuO NPs. (Experimental conditions: DV31 concentration 25 mg/L; 0.5 mL H_2_O_2_; sample volume 100 mL; 10.0 mg CuO NPs; 30 min contact time and temperature at 25 °C).

The solution pH is another principal parameter which controls the removal process, especially for DV31 dye, because they contain different functional groups, amide group (HN-CO-R) and amino group (HN-R), which may ionize depending on the pH of the solution. The effect of solution pH on DV31 dye degradation by G CuO NPs was therefore explored in the pH range from 4.0 to 12.0. The results are presented in Figure 11 and it was observed that the removal percentage increased from 41.15% to 59.27% as the pH of the solution changed from 4.0 to 8.0. Further increase in the solution pH from 8.0 to 12.0 was accompanied by a sharp decrease in the removal percentage from 59.27% to 10.11%. This phenomenon could be due to many factors. Firstly, the point-of-zero-charge of CuO NPs is equal to 9.0 [42,43]; which means that CuO NPs are positively charged below a pH value of 9.0 and negatively charged above a pH value of 9.0; this is because at pH less than 7.0 the major abundant species is [H^+^], while at pH more than 7.0 the major abundant species reacts with [OH^-^], which adsorbed at the CuO surface. At the same time, DV31 is characterized by a p*Ka* value of 4.0. As the pH value increased from 4.0 to 9.0, the removal of DV31 increased due to the attraction forces between the positively charged G CuO NPs surface and the negatively charged DV31 dye molecules, which agreed well with a previous study [44]. Further increase in the solution pH from 8.0 to 11.55 was associated with a sharp decrease in the removal percentage of DV31, due to the electrostatic repulsion between the negatively charged G CuO NPs and the negatively charged DV31 molecules. This may indicate that the adsorption and further degradation of DV31 on G CuO NPs surface could be electrostatic-in-nature and mainly depends on the charge of both DV31 dye molecules and the G CuO NPs surface.

### 3.3. Kinetic Studies

To understand the adsorption/removal of pollutants such as DV31 dye from an aqueous solution by a solid catalyst/adsorbent such as G CuO NPs, it must be studied kinetically in order to obtain the appropriate mathematical models to describe the interactions between the pollutant and the solid adsorbent, in order to design suitable adsorbent materials for environmental and industrial applications. Figure 9 shows the adsorption/removal experimental data representing the variation of the amount DV31 dye adsorbed/removed from aqueous solution by G CuO NPs (q_t_) with time and Figure 8 shows that the adsorption/removal of DV31 by G CuO NPs reached equilibrium within 45 min. Because all chemical reactions involving solid reactants such as the CuO NPs are usually based on the adsorption of the reactants in aqueous solution on the surface of the solid reactants, it is very important to apply the adsorption kinetic models to the removal experimental data. The adsorption experimental data were treated kinetically using the most common and well-known kinetic models to understand the nature of the adsorption process; Lagergren pseudo-first-order kinetic model [45] and pseudo-second-order kinetic model [46].

**Figure 12:**
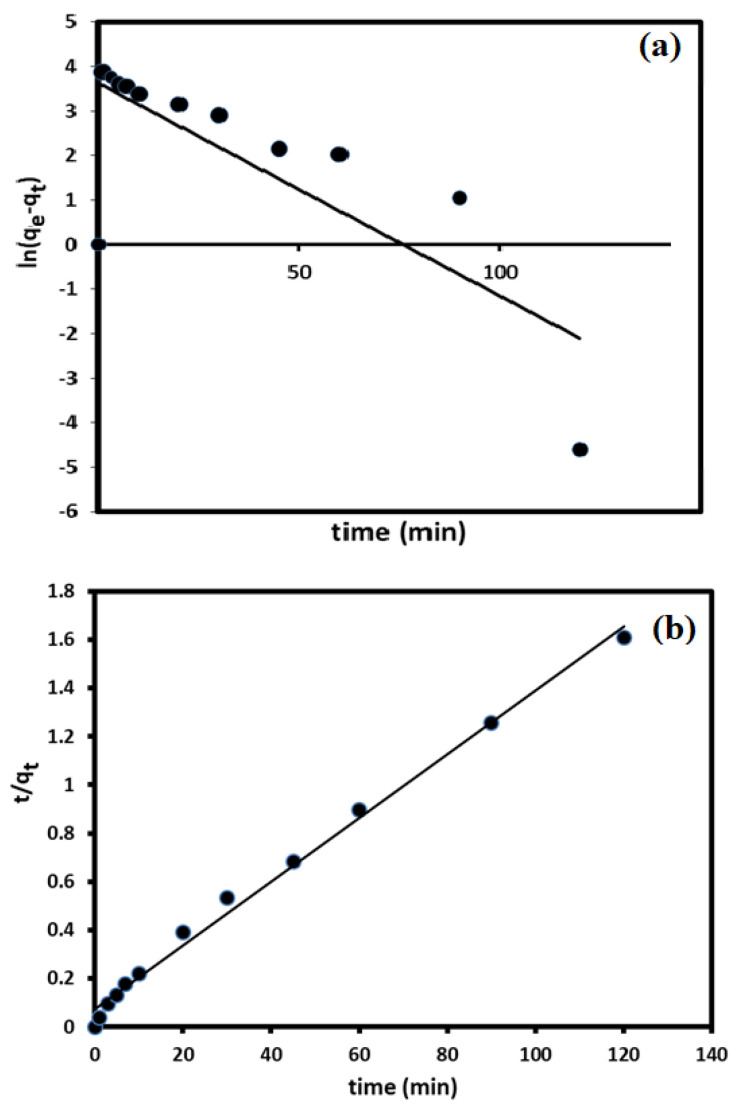
Pseudo-first-order kinetic model plot (**a**) and pseudo-second-order kinetic model plot (**b**) for DV31 dye removal from an aqueous solution by G CuO NPs. (Experimental conditions: DV31 concentration 25 mg/L; 1 mL H_2_O_2_; sample volume 200 mL; 20.0 mg CuO NPs; pH 8.6 and temperature at 25 °C).

Lagergren pseudo-first-order kinetic model is one of the most frequent kinetic models used to describe the adsorption of different adsorbates from an aqueous solution by solid adsorbent [45,47] and it is linearized for as follows:

(4) $$\ln\left(q_{e}-q_{t}\right)=\ln q_{e}-k_{1}t$$

where k_1_ (min^−1^) is the pseudo-first-order rate coefficient and q_e_ and q_t_ are the values of the amount adsorbed/removed per unit mass at equilibrium and at any time t, respectively. Applying Equation (4) to the experimental adsorption data in Figure 12a and plotting ln (q_e_ − q_t_) vs. t did not give a straight line, and low convergence and an unacceptable R^2^ value were obtained, as is shown in Figure 12a and Table 1, demonstrating the unsuitability of the Lagergren pseudo-first-order kinetic model for describing the adsorption/removal of DV31 dye by G CuO NPs from aqueous solution.

The linearized equation of the pseudo-second-order kinetic model equation is given as:

(5) $$\frac{t}{q_{t}}=\frac{1}{k_{2}q_{e}^{2}}+\frac{t}{q_{t}}$$

where k_2_ (g/(mg·min)) is the pseudo-second-order rate coefficient. Applying the pseudo-second-order kinetic model equation to the adsorption/removal experimental data in Figure 8, by plotting of t/q_t_ and t of Equation (5), converges well, with excellent R^2^ value, and a straight line was obtained from which q_e_ and k_2_ were estimated from the slope and intercept of the plot, respectively, as presented in Figure 11b and Table 1, confirming the appropriateness of the pseudo-second-order kinetic model for the description of DV31 dye adsorption/removal by the G CuO NPs from aqueous solution.

Another test was performed to confirm the suitability of the pseudo-second-order kinetic is by calculating the Chi-square test using the following mathematical Equation (6) [48] and Sum of the squares of errors (SSE) at Equation (7) [49]:

(6) $$\chi^{2}=\sum \frac{{\left(q_{e,\mathrm{calc}}-q_{e,\exp}\right)}^{2}}{q_{e,\mathrm{calc}}}$$

(7) $$\mathrm{SSE}=\overset{n}{\underset{i=1}{\sum }}{\left(q_{e,\mathrm{calc}}^{i}-q_{e,\exp}^{i}\right)}^{2}$$

where q_e,calc_ and q_e,exp_ are the calculated and experimental removed amount of DV31 dye molecules per unit mass of the G CuO NPs at equilibrium. The χ^2^ values were 35.84 and 0.02284 and the achieved SSE values were 1354 and 1.734, for the pseudo-first-order kinetic model and the pseudo-second-order kinetic model applied, respectively, indicating the appropriateness of the pseudo-second-order kinetic model for describing the removal process.

Based on the above results, it could be concluded here that the pseudo-second-order kinetic model was the best for describing the adsorption of DV31 dye from aqueous solution by CuO NPs.

The adsorption usually occurs in four distinctive steps: bulk diffusion, mass action, liquid film diffusion and finally intra-particle diffusion, which is classified as a pore and surface diffusion. This part focuses on the determination of the rate-determining step for the adsorption/diffusion process.

**Figure 13:**
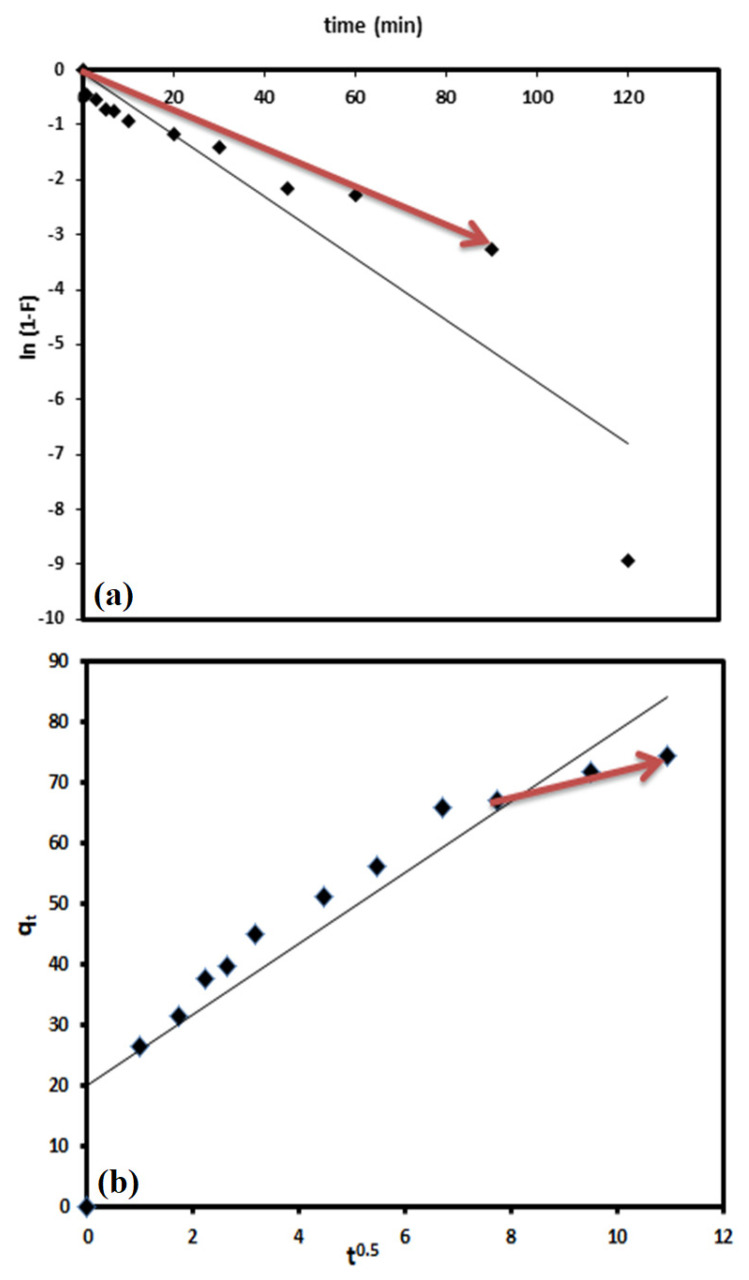
Liquid film diffusion plot (**a**) and intraparticle diffusion plot (**b**) for the removal of DV31 dye from an aqueous solution by G CuO NPs. (Experimental conditions: DV31 concentration 25 mg/L; 1 mL H_2_O_2_; sample volume 200 mL; 20.0 mg CuO NPs; pH 8.6 and temperature at 25 °C).

The linearized form of the liquid film diffusion equation is as follows [50]:

(8) $$\ln\left(1-F\right)=-k_{\mathrm{fd}}t\;$$

where F is the fractional attainment of equilibrium and equals to $\frac{q_{t}}{q_{e}}$ and k_fd_ (min^−1^) is the film diffusion rate coefficient. By applying Equation (8) to the experimental data in Figure 9 and by plotting ln (1 − F) versus t, a straight line was obtained with zero intercept, but only for the first 90.0 min of the interaction, from which k_fd_ was estimated from the slope of the plot, as shown in Figure 13a, for the removal of DV31 dye by G CuO NPs, with acceptable R^2^ value of 0.965 for the first 90.0 min of the adsorption/removal process.

The linearized intraparticle diffusion equation is as follows [50]:

(9) $$q_{t}=k_{\mathrm{id}}t^{0.5}+C_{i}$$

where k_id_ (mg/(g.min^0.5^)) is the intraparticle diffusion coefficient and C_i_ (mg/g) is related to the boundary layer thickness. Applying Equation (9) to the experimental data by plotting qt versus t^0.5^, as presented in Figure 12b, a straight line was obtained for the removal of DV31 dye by CuO NPs after the first 60.0 min of the interaction with a good R^2^ value of 0.99. This may indicate that the removal of DV31 dye by the G CuO NPs was controlled by both liquid film and intraparticle diffusion kinetics during the time of adsorption.

### 3.4. Thermodynamic Studies

Thermodynamic parameters; enthalpy change (ΔH), entropy change (ΔS) and Gibbs free energy change (ΔG), were calculated to evaluate the thermodynamic feasibility and the spontaneous nature of the removal of DV31 dye molecules by G CuO NPs from aqueous solution. Thermodynamic parameters were calculated using the variation of the thermodynamic distribution coefficient D with a change in temperature according to the equation [51]:

(10) $$K_{d}=\;\frac{q_{e}}{C_{e}}\times \;1000$$

where q_e_ is the amount of DV31 molecules adsorbed/removed by G CuO NPs (mg/g) at equilibrium and C_e_ is the equilibrium concentration of DV31 in solution (mg/L). The ΔH and ΔS could be calculated according to the following equation [52]:

(11) $$\ln K_{d}=\;\frac{\Delta S}{R}-\frac{\Delta H}{RT}$$

**Figure 14:**
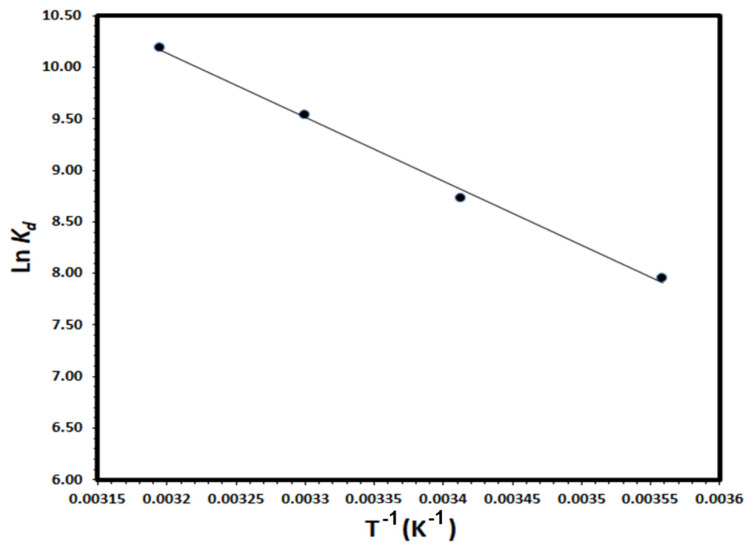
Plot of ln K_d_ vs. T^−1^ for the thermodynamic parameters calculations for DV31 adsorption from an aqueous solution by G CuO NPs. (Experimental conditions: DV31 concentration 25 mg/L; 0.5 mL H_2_O_2_; sample volume 100 mL; 10.0 mg CuO NPs; 10 min contact time and pH 8.6).

A straight line was obtained by plotting ln K_d_ vs. 1/T; as shown in Figure 14 and the ΔH and ΔS values were calculated from the slope and the intercept of the straight line, respectively. The ΔG values were calculated at 298K from the relation:

ΔG = ΔH − TΔS(12)

Thermodynamic parameters for the adsorption/removal of DV31 molecules degraded by CuO NPs were calculated and ∆H, ∆S and ∆G values were +51.64 kJ/mole, +249.5 J/mole.K and −22.71 kJ/mole, respectively. The positive value of the enthalpy change indicated the endothermic nature of DV31 by G CuO NPs and the positive value of the entropy change indicated the increase in the degree of freedom at the solid-liquid interface and apparently, the negative value of the free energy change as would be expected for a product favored and spontaneous degradation process. Accordingly, the negative value of ∆G and positive values of ∆H and ∆S suggested that the adsorption of DV31 by CuO NPs from an aqueous solution is an entropy-driven process.

### 3.5. The Removal Mechanism

**Figure 15:**
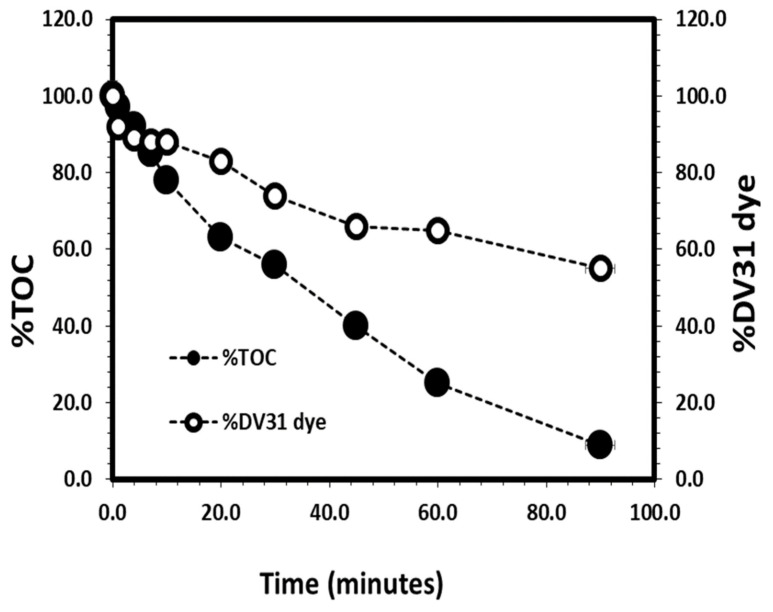
Variation of the %DV31 dye and %TOC in water with time for the removal of DV31 dye by CuO NPs from aqueous solution. (Experimental conditions: DV31 concentration 25 mg/L; 2 mL H_2_O_2_; sample volume 400 mL; 40.0 mg CuO NPs; pH 8.6 and temperature at 25 °C).

The reaction between G CuO NPs and H_2_O_2_ is well known and studied as an AOPs mechanism, CuO NPs react with H_2_O_2_ and produce OH•, which attacks nearly all organic complexes. Therefore, the reaction of the organic pollutant with the HO• leads to a complete breakdown of the organic compound and, as a result, AOPs diminish the concentration of the pollutant from a few hundred mg/L to less than 5 ng/L. Many studies have shown that, with AOP, organic chemicals disintegrate and become smaller and easily biodegradable [53,54]. The degradation of DV31 dye molecules through AOPs was monitored through TOC analysis. The %TOC, total organic carbon, in the solution with time were measured during the catalytic degradation process of the DV31 dye by G CuO NPs as shown in Figure 15. It is observed that the decrease in the % DV31 dye in solution was associated with the same decrease trend of the %TOC in solution. The DV31 dye in solution decreased to around 88% within 10 min associated with %TOC in the water of 78% and this percentage was decreased to 9% after 90 min with a %DV31 dye in the solution of 55%. This may indicate the successful removal of the DV31 dye by G CuO NPs in presence of H_2_O_2_.

### 3.6. Environmental Applications

Application of the proposed heterogeneous catalyst; G CuO NPs, for the removal of DV31 dye from real samples is a crucial point to validate the applicability of the method. Therefore, the removal of DV31 dye by CuO NPs from three different samples collected from different environments was investigated, namely, well water sample collected from a well, a seawater sample collected from Red Sea in Jeddah city, wastewater sample collected from King Abdulaziz University Wastewater (KAUWW). The % removed was calculated by comparing DV31 dye concentrations before and after the addition of CuO NPs in presence of hydrogen peroxide. The % DV31 dye removed from the real sample was calculated and was found to be 100% in all real samples. This may indicate that G CuO NPs are a promising and potential adsorbent for the remediation of a polluted environment.

## 4. Conclusions

This research work explored the preparation of CuO NPs via eco-friendly and green method using the extract of *corchorus olitorus* leaves (Molokhaia) and their characterization using XRD, TEM, XPS and textural properties and the results demonstrated the efficacious formation of monoclinic tenorite CuO nanoparticles with average particle size of 12 nm and surface area of 11.1 m^2^/g. The eco-friendly green CuO NPs were used for the environmental remediation for the efficient catalytic degradation of direct violet dye (DV31) via advanced oxidation process (AOP) in presence of H_2_O_2_. The impact of AOP environmental parameters affecting the degradation process was investigated and the results revealed that most of the DV31 were removed from the solution using G CuO NPs within 45 min at pH 8.0 and 298 K. Moreover, the catalytic degradation of the direct violet dye using the ecofriendly green CuO NPs was studied kinetically and thermodynamically and the results showed that the catalytic degradation process agreed well with the pseudo-second order kinetic model with removal capacity of 74.59 mg/g. Additionally, the process was spontaneous; (∆G = −22.71 kJ/mole), endothermic in-nature (∆H = +51.64 kJ/mole), with increase in the degree of freedom at the solid-liquid interface (∆S = +249.5 J/mole.K), suggesting that the adsorption/removal of DV31 by G CuO NPs from an aqueous solution is an entropy-driven process. Finally, the catalytic degradation of the direct violet dye using G CuO NPs was studied in real water samples and the results revealed the outstanding performance of the G CuO NPs and their possible application for the remediation of a polluted environment.

## Figures and Tables

**Table 1:** Different kinetic models’ parameters for DV31 removal from an aqueous solution by G CuO NPs.

Parameter	Kinetic Model	
	PFO	PSO
K	0.0479	0.0024
q_e,exp_ (mg/g)	74.59	74.59
q_e,calc_ (mg/g)	37.79	75.90
R^2^	0.603	0.994
χ^2^	35.84	0.02284
SSE	1354	1.734

## Data Availability

Not applicable.
